# Perpetuation of household food insecurity during COVID-19 in South Africa

**DOI:** 10.1186/s41043-023-00441-y

**Published:** 2023-09-12

**Authors:** Nicole De Wet-Billings

**Affiliations:** Demography and Population Studies, Schools of Social Sciences and Public Health, University of the Witwatersrand, Johannesburg, South Africa

**Keywords:** Food insecurity, COVID-19, South Africa, Inequality, NIDS CRAM survey

## Abstract

**Background:**

Perpetual food insecurity has long-term health and development effects on populations. The global pandemic created sub-populations that were newly food insecure, but there exists sub-populations were food insecure, and COVID-19 held that situation. This study seeks to identify the demographic and socioeconomic characteristics of the perpetually food insecure in South Africa in order to obtain specific evidence of populations to be prioritised in the post-pandemic era.

**Methods:**

Secondary data from the South African National Income Dynamics CRAM Survey for rounds (Waves) 1 and 5 are analysed. The study population are those respondents who reported a household member not having enough food to eat in the early stages of the pandemic (1st round) and remained without sufficient food a year later (5th round). The study controls for the demographic and socioeconomic characteristics of the population but also changes to employment status, social grant access and willingness to be vaccinated. Descriptive and analytical statistical tests are used.

**Results:**

A total of 26.15% of respondents were food insecure at the start of the pandemic. Of these, 41.09% remained food insecure a year later. The drivers of perpetual food insecurity during the pandemic include unemployment (OR = 2.09; CI 1.335293–3.265678), still being unemployed (OR = 1.86; CI 1.308032–2.636252), seven or more (≥ 7) household members (OR = 1.24; CI 1.1611329–1.610126), those with only a primary education (OR = 1.11; CI 1.5051066–2.434695), participants between the ages of 45 and 64 years old (ORs = 1.03 and 1.20; CIs 1.0171956–1.0171956 and 1.1733304–2.144875, respectively) and women (OR = 1.09; CI 1.0745444–1.406035).

**Conclusions:**

South Africa needs to address socioeconomic challenges and inequalities to assist the perpetually food insecure and to ensure that, should there be a pandemic resurgence, or a new pandemic, individuals and households in the country are in a better financial situation and appropriately supported to avoid food insecurity at all costs.

## Background

The COVID-19 pandemic has worsened levels of poverty and inequality [1, 2]. Across the world, massive amounts of deaths, job losses and disruptions to education have led to economic, social and health care pressures. No country has been exempt, and the world is yet to fully understand the long-term consequences of the virus. However, prior to the pandemic, economies and populations in the global south were already battling poverty and inequality. Among the more specific challenges has always been food insecurity. This is defined as the lack of access to enough food for an active and healthy life and extends to include that is healthy, nutritious and culturally appropriate that is consistent [3, 4].

For many lower-to-middle-income countries (LMICs) food insecurity is an outcome of climate change and natural disasters, conflict, income inequality, farming skills shortages and land grabs, among others [5–9]. Also in sub-Saharan Africa, the majority of food is produced by smallholder farms and agricultural systems that have the potential to increase employment and raise local economies but are particularly vulnerable to poverty, market fluctuations and climate change [10–12]. The impact of the COVID-19 on food production in the region, only made the situation worse. Research shows that in Southern Africa between 9.1 and 41.7% of farmers had challenges in accessing labourers needed to work during the early stages of the pandemic, and between 27.6 and 41.7% had problems accessing credit or financing to sustain output [13]. This resulted in the closure of many smallholder farms and the impact affected not only the farmers and their households, but the many impoverished households where food was being distributed [14].

Perpetual or long-term food insecurity has a negative impact on the health, ability to complete education and/or engage in labour market activities and other development prospects for affected populations. In particular, studies have shown an association between food insecurity and malnutrition, with young children and the elderly being the most affected; increased health care expenditure by individuals and governments and poor mental health outcomes including depression [15–17]. Among children and adolescents, food insecurity is associated with diminished learning capacity and an increased inability to pay attention in school [18]. For adults, not having sufficient and consistent good food is a risk factor for unemployment and risky behaviours such as transactional sexual intercourse and illicit drug use [19–21].

Prior to the pandemic, approximately 20% of South African households reported not having enough food to eat in the last 7 days [22]. This percentage increased to over 25% during the early months of the pandemic [23]. The ‘hard lockdown’ in the country which saw an almost complete standstill to all social and economic activities, with the exception of essential services (mostly in the health care sector) lasted for approximately 4 months, and because many businesses closed or suffered due to lack of activity, about 22% of adult females and 10% of adult males living in South Africa became unemployed [24]. To buffer this, the government increased the value of some social grants and introduced a new grant to households whose members were already unemployed or lost employment due to the lockdown restrictions. The value of this new ‘social relief of distress grant’ was not much at USD 23 (ZAR 350) and with the current mean number of household members at five per household, it is clear to see why food insecurity numbers increased in the country [25, 26].

Households suffering from food insecurity prior to the pandemic, may have been relieved to receive this additional grant and this may have lifted them out of a dire situation. But of particular concern are the households unable to escape food insecurity during the pandemic. This study aims to identify and analyse the social and economic determinants of perpetual household food insecurity during the height of the COVID-19 pandemic in South Africa.

## Methods

### Data

The data used for this study comes from the National Income Dynamics CRAM survey of 2020 to 2021. The NIDS CRAM consists of five waves or rounds of data starting in May 2020 until May 2021. The rounds of data collection did not coincide with the waves of the pandemic. For this reason, this study refers to the ‘waves’ of data collection as ‘rounds’. For this study, rounds 1 and 5 are used to measure the outcome of perpetual food insecurity. The NIDS CRAM collected data on social, economic and health challenges experienced by the population in South Africa during the height of the COVID-19 pandemic. Specific questions related to the pandemic including its disruption to school, work and livelihoods were also included [27].

### Study population and sample

**Fig. 1:**
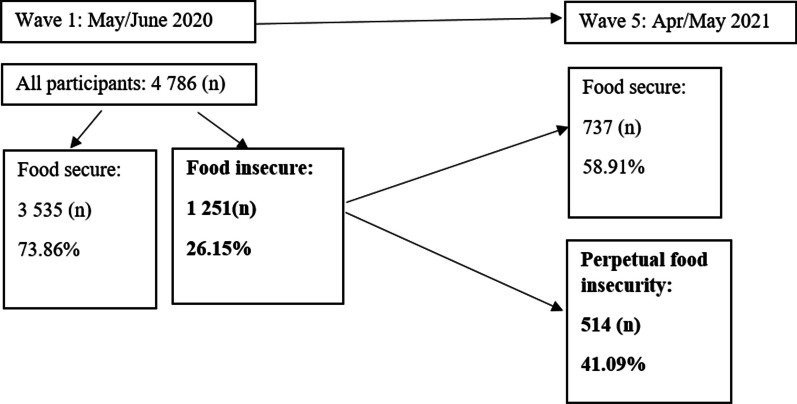
Frequency and percentage distribution of food security status from the 1st round to 5th round

Figure 1 shows that in the 1st round, the sample consisted of 4786 participants. From this, 73.86% answered ‘no’ to the question on any household member not having sufficient food to eat in the 7 days prior to the interview and are therefore considered ‘food secure’. These participants were dropped from the analysis and the 26.15% (*n* = 1251) participants reporting at least one household member not having enough food to eat in the 7 days prior to the interview, or ‘food insecure’, were kept in the analysis.

In the 5th round, the (*n*) 737 (58.91%) participants whose status changed from being food insecure to secure, as indicated by a negative (no) answer to the same question regarding any household member not having sufficient food to eat in the 7 days prior to the interview, posed a year later, were excluded from the analysis. The 41.09% (*n* = 514) who responded positively a second time to any household member not having sufficient food to eat in the 7 days prior to the interview were kept in the analysis and are here considered perpetually food insecure due to being food insecure in both waves of data.

### Study variables

#### Dependent variable

The dependent variable for this study is ‘perpetual food insecurity’ and is measured as the number of respondents who had at least one member of the household not have enough food to eat in the 7 days prior to the interview at the start of the 1st round and continued into the 5th round. To create this variable, participants coded as ‘food insecure’ in round 1 were carried through to round 5, 1 year later, and if they still responded ‘yes’ to the fore mentioned question, were coded as ‘perpetual food insecurity’ [1]. If the respondent’s food security status changed they were coded as ‘no perpetual food insecurity’ (0).

#### Independent variables

The demographic and socioeconomic status of respondents at the 1st round were included in the study. Some of these variables would not change a year later, for example, gender and population group. And some variables would not have significantly changed, such as age-group, which is in 10 years age intervals. Other variables unlikely to have changed due to the lockdown restrictions on movement are, economic and social activity including education status (also in broad intervals), number of household members, type of dwelling, access to a social grant and access to water and/or electricity. There are however some variables that would have changed and are estimated using both round 1 and round 5 data and these include employment status change, access to the Social Relief Distress Grant (which only became available during 2020) and vaccine willingness, which was only asked in the 5th round. These variables are collectively called the ‘participant pandemic impact’ variables in this study.

#### Statistical analysis

**Fig. 2:**
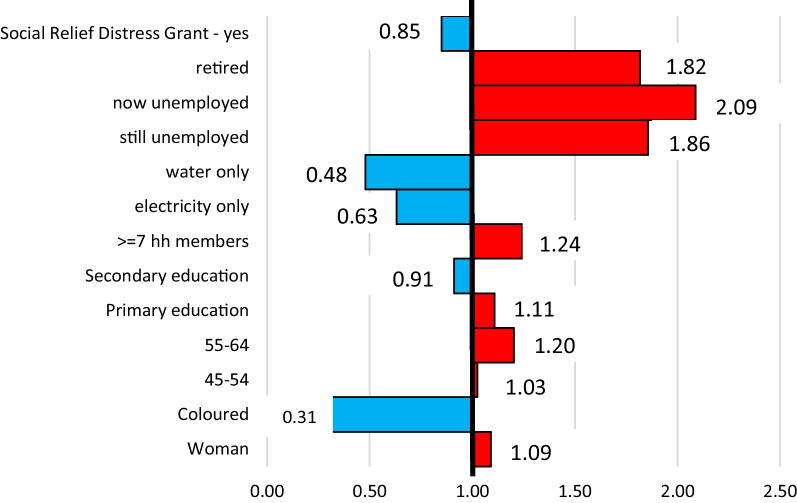
Logistic regression results showing the odds of perpetual food insecurity (in both Wave 1 and Wave 5) by all participant characteristics

Cross-tabulation has been used to describe the distribution (frequency and percentage) of food insecurity in the 1st round and perpetual food insecurity by the various independent variables. Chi-square analysis was done to establish the relationship between variables, and *p* values are reported. A binary logistic regression model was fit to the data with the outcome of perpetual food insecurity (yes = 1 and no = 0) by all variables in the study and is seen in Fig. 2 of the results. This model combines the demographic, socioeconomic and pandemic-related variables in a single model with the round 5, perpetual food insecurity outcome.

## Results

In total, 26.11% of participants were in food insecure households (yes) at the start of the pandemic in 2020, at the 1st round (Table 1). By gender of the study participants, 26.24% of women and 25.91% of men were food insecure. Among the population groups in the country, 28.41% of African participants were food insecure compared to only 3.86% of White respondents. The results in Table 1 further show that more 35–44 year olds (30.36%) were food insecure at the start of the pandemic than any other age group. However of concern is that almost 20% of youth (18–24 years old) and 18.43% of the elderly (65 + years old) were also food insecure at this time. By highest level of education almost 30% of respondents with no education and 34.44% of those with only a primary education were food insecure. Further 28.7% of unemployed participants were in food insecure households. Almost 30% of respondents in households with seven or more (≥ 7) members, 35.95% who reside in traditional dwellings and 25.99% not accessing a social grant were also food insecure during this period. Finally, 33.80% of participants with electricity only, 33.10% with neither water nor electricity, 30.85% with water only and 23.61% with access to both water and electricity were food insecure at the start of the pandemic.

**Table 1:** Percentage distribution of participant demographic and socioeconomic characteristics by food insecurity in the 1st round of data collection

Respondent characteristics	Food insecure				
	Yes		No		Total
	*n*	%	*n*	%	*n*
Total	1249	26.11	3534	73.89	4783
Gender*					
Man	469	25.91	1341	74.09	1810
Woman	780	26.24	2193	73.76	2973
Population group*					
African	1170	28.41	2948	71.59	4118
Coloured	70	16.71	349	83.29	419
Asian/Indian	3	8.57	32	91.43	35
White	8	3.86	199	96.14	207
Other	0	0	7	100	7
Age group*					
18–24	130	19.79	527	80.21	657
25–34	334	26.61	921	73.39	1255
35–44	371	30.36	851	69.64	1222
45–54	225	27.21	602	72.79	827
55–64	117	27.46	309	72.54	426
65 +	73	18.43	323	81.57	396
Highest level of education*					
None	33	29.46	79	70.54	112
Primary	239	34.44	455	65.56	694
Secondary	979	24.6	3001	75.4	3980
Employment status*					
Employed	167	16.55	842	83.45	1009
Unemployed	1084	28.7	2693	71.3	3777
Number of household members*					
≤ 6	825	24.58	2531	75.42	3356
≥ 7	426	29.81	1003	70.19	1429
Type of dwelling*					
House	880	23.54	2859	76.46	3739
Traditional	197	35.95	351	64.05	548
Informal/other	174	34.87	325	65.13	499
Accessing a social grant*					
Yes	267	26.7	733	73.3	1000
No	984	25.99	2802	74.01	3786
Access to water and electricity*					
Water only	29	30.85	65	69.15	94
Electricity only	336	33.80	658	66.20	994
Both	839	23.61	2715	76.41	3553
Neither	47	33.10	95	66.90	142

^*^p values < 0.05

Table 2 shows the frequency and percentage distribution of all participant characteristics by perpetual food insecurity outcomes, which is food insecure from the 1st to the 5th round of data collection. By gender, 42.69% of women and 38.59% of men were still food insecure. According to population group 42.48% of African and 37.50% of White participants remained food insecure during the period. Among the various age-groups, over 40% of 35 to 64 year olds remained food insecure as did 42.31% of 18 to 24 year olds. Participants with no education (45.45%) and those with only primary education (46.44%) had the highest levels of perpetual food insecure during the period. Again, large households (≥ 7 members) (46.24%), traditional dwellings (49.24%) and those accessing at least one social grant (41.57%) also reported the highest levels of perpetual food insecurity. Almost 59% of participants residing in household with water only, 48.21% of those with electricity only, and 46.81% with neither water nor electricity remained food insecure in the period.

**Table 2:** Percentage distribution of all participant characteristics by perpetual food insecurity

Respondent characteristics	Perpetual food insecurity—both in rounds (1st and 5th)				
	Yes		No		Total
	*n*	%	*n*	%	*n*
Total	514	41.15	737	59.01	1249
Gender*					
Man	181	38.59	288	61.41	469
Woman	333	42.69	447	57.31	780
Missing	0	0.00	2	100.00	2
Population group*					
African	497	42.48	673	57.52	1170
Coloured	13	18.57	57	81.43	70
Asian/Indian	1	33.33	2	66.67	3
White	3	37.50	5	62.50	8
Age group*					
18–24	55	42.31	75	57.69	130
25–34	124	37.13	210	62.87	334
35–44	154	41.51	217	58.49	371
45–54	98	43.56	127	56.44	225
55–64	56	47.86	61	52.14	117
65 +	26	35.62	47	64.38	73
Highest level of education*					
None	15	45.45	18	54.55	33
Primary	111	46.44	128	53.56	239
Secondary	388	39.63	591	60.37	979
Number of household members*					
≤ 6	317	38.42	508	61.58	825
≥ 7	197	46.24	229	53.76	426
Type of dwelling*					
House	342	38.86	538	61.14	880
traditional	97	49.24	100	50.76	197
informal/other	75	43.10	99	56.90	174
Accessing a social grant*					
Yes	111	41.57	156	58.43	267
No	403	40.96	581	59.04	984
Access to water and electricity*					
Water only	17	58.62	12	41.38	29
Electricity only	162	48.21	174	51.79	336
Both	313	37.31	526	62.69	839
Neither	22	46.81	25	53.19	47

*p values < 0.05

Table 3 shows the percentage distribution of ‘participant pandemic impact’ characteristics by perpetual food insecurity or being food insecure in both rounds of data collection (1 and 5). In total, 41.09% of participants were still food insecure in the 5th round. Those who remained unemployed (45.63%), became unemployed (48.03%) and were retired (42.22%) during the period had higher rates of perpetual food insecurity. Of the participants who were accessing the Social Relief Distress Grant (yes) 41.36% were perpetually food insecure and 41.95% of respondents willing to be vaccinated also remained food insecure.

**Table 3:** Percentage distribution of ‘participant pandemic impact’ characteristics by perpetual food insecurity

Participant pandemic impact characteristics	Perpetual food insecurity—both in rounds (1^st^ and 5^th^)				
	Yes		No		Total
	*n*	%	*n*	%	*n*
Total	514	41.09	737	58.91	1251
Employment change*					
Still employed	65	29.95	152	70.05	217
Still unemployed	277	45.63	330	54.37	607
Now unemployed	73	48.03	79	51.97	152
Retired	57	42.22	78	57.78	135
Now employed	42	30	98	70	140
Social relief distress grant*					
No	313	40.92	452	59.08	765
Yes	201	41.36	285	58.64	486
Vaccine willingness*					
Yes	396	41.95	548	58.05	944
No	107	38.08	174	61.92	281

*p values < 0.05

The results of the adjusted logistic regression model, showing odds ratios are presented graphically in Fig. 2. Only the statistically significant (*p* values < 0.05) results are shown. From the figure, the likelihood of remaining food insecure during the pandemic are higher for retired (OR = 1.82; CI 1.040552–3.175925), now unemployed (OR = 2.09; CI 1.335293–3.265678), still unemployed (OR = 1.86; CI 1.308032–2.636252), seven or more (> = 7) household members (OR = 1.24; CI 1.1611329–1.610126), those with only a primary education (OR = 1.11; CI 1.5051066–2.434695), participants between the ages of 45 and 64 years old (ORs = 1.03 and 1.20; CIs 1.0171956–1.0171956 and 1.1733304–2.144875, respectively) and women (OR = 1.09; CI 1.0745444–1.406035).

The odds of being food insecure are less for those accessing the Social Relief Distress Grant (OR = 0.85: CI 0.6633155–0.989399), those with water (OR = 0.48: CI 0.218738–0.646399) or electricity (OR = 0.63; CI 0.2835275–0.805429) only, participants with a secondary education (OR = 0.91: CI 0.04177126–0.989031) and coloured (OR = 0.31; CI 0.1621159–0.6025793) respondents.

## Discussion

The long-term impact of the COVID-19 pandemic is yet to be fully realised. Food insecurity, a challenge prior to, was worsened during the pandemic [28–30]. The results of this study are important for two reasons. First, the study highlights the extent of food insecurity during the pandemic particularly among those who were already affected. This is useful to policies and programmes in South Africa that aim to rectify or correct worsened situations caused by the virus. Second, the results of the study can be used beyond addressing COVID-19 and are useful to other infectious disease epidemics and the future planning for food security during these worsened times.

Perpetual food insecurity during the COVID-19 pandemic affected about 41% of participants who were already food insecure. South Africa’s historic and current economic difficulties have resulted in worsening inequality and poverty. Prior to the pandemic, the national unemployment rate was 25.54% in 2019, and this rose to 28.77% in 2021 and is currently at 32.7% [31, 32]. Some of the causes of the economic decline in the country were present before COVID-19 and these include a weakened education system, slow structural growth, increasing inflation and an increasing reliance on social assistance grants [33, 34]. However, the pandemic worsened the state of these challenges in the country, therefore creating an economic downfall that could not be predicted and the full extent of the consequences are yet to be realised. Therefore, it is not just food insecurity that became a continuous problem, but related to this is the perpetuation of poverty and inequality in South Africa.

This study considered only the perpetuation of food insecurity and not the individuals or households that became food insecure due to the pandemic. That is, the newly food insecure households. However, other studies have found that this did in fact occur with approximately 30% of households experiencing food insecurity for the first time during the pandemic [35].

Women are worst affected by food insecurity and the perpetuation of food insecurity. This study showed a higher percentage of women in the early stages of the pandemic as well as a year later being food insecure compared to their male counterparts. This result is not surprising in South Africa where women are disproportionately affected by poverty and inequality. Recent statistics reflect historical patterns that show females having lower employment rates (48% compared to 52% for males), lower levels of tertiary education (approximately 48%), higher rates of HIV and AIDS (26.3%) and high rates of gender-based violence of between 25 and 40% of South African women have experienced sexual and/or physical IPV in their lifetime [32, 36, 37]. Within this context, it is almost impossible for women to not experience food insecurity. The result of this study is concerning however in light of the findings that about 18% of adult women in South Africa are household heads and among these households about 33% have only the female member employed [38]. Household food insecurity means that more than one, if not all, members of a household do not have access to sufficient food. The knock-on effects of this relate to child health and development as well as care for the elderly of whom women are the primary caretakers.

The study showed that few respondents were able to gain new employment during the pandemic, while about only 17% were able to retain their employment, yet the majority of respondents remained unemployed or were retired. This is not encouraging for households that were already food insecure. For those who were able to maintain or find employment, spending wages on food would have been in competition with paying for other living expenses and transport during the period. An analysis of household spending in South African households found that food expenditure is still the largest household expense, with costs increasing, but also the increased cost of housing, electricity, fuel and transport are meaning that South Africans are unable to purchase sufficient and health food to sustain themselves and their households [39].

Poor socioeconomic status remains the driving force behind insecurity. The current study has found that unemployment, not having both water and electricity, large household sizes and less than a tertiary education are all associated with an increased possibility that households will remain perpetually food insecure in South Africa. Also, and importantly for this specific group of individuals, having access to the Social Relief Distress Grant is associated with lesser odds of being food insecure. A study in South Africa that examined child hunger and food security in the same period as the current study argue that the reduction in emergency relief along with the constrained economic situation, are likely to result in food insecurity and hunger remaining high in the country post-pandemic period [40]. To date, there is evidence that this is true, with a most recent study showing that food insecurity in South Africa is at its highest since the democratisation of the country in 1994 [41]. Added to this research, others suggest that only with massive and dramatic economic reform and an urgent end to the energy crisis in the country, will there be a chance for the better livelihood outcomes [42, 43].

There are a few strengths to the current study. First, the design allows for robust analysis of causal factors contributing to food insecurity that could be adopted in later studies or with other longitudinal data. This is beneficial to countries like South Africa where there is a constant need to research sustainable livelihoods of the population, and COVID-19 might not be the only global crisis to have an effect on local or regional economies in the future. Second, the results of the study point to an extremely vulnerable population, who are often grouped more broadly under the banner of poverty. However, this group of perpetually food insecure population require specific attention as their state suggests a structural inability to secure sufficient food to eat. Finally, this study uses recent data on COVID-19 that was collected thoroughly and in a timeous manner, making these results locally relevant and at the right time to be used in policy and programme reformations in the country.

The study is also subject to limitations. First, food insecurity is not measured in the interim of the 12-month time frame of the study. Since the time frame of the study however is only 12 months, and there were no significant changes during the period, especially since the country’s lockdown was still enforced during the period, there would not have been major changes to employment or education status. Thus not having interim or mid-point data is not a major flaw of the study design and did not impact the results. Second, the study cannot determine how much household income and grants were spent on food during the pandemic so it is not known if food expenditure was a priority in insecure households. This would have placed the status of perpetuation in a context of spending behaviour which would be useful explain the results.

## Conclusion

In conclusion, perpetual food insecurity, as a result of the pandemic, is a threat to the health and development of individuals in affected households. This is in addition to socioeconomic standards in the country not improving in substantive ways. The pandemic worsened food security outcomes, particularly for those already affected, but with prioritising socioeconomic reform, South African households will continue to lack sufficient food to live healthy and meaningful lifestyles in the future. As such, to lift currently affected households out of precarious food security situations and to avoid more households becoming food insecure, due to future pandemics or not, the country must prioritise the development (education, employment, etc.) of women and the poor (unemployed, large households) in the country.

## Data Availability

The data are freely downloadable from https://cramsurvey.org/.
